# Proposal for a method to estimate nutrient shock effects in bacteria

**DOI:** 10.1186/1756-0500-5-422

**Published:** 2012-08-08

**Authors:** Nuno F Azevedo, Sofia M Bragança, Lúcia C Simões, Laura Cerqueira, Carina Almeida, Charles W Keevil, Maria J Vieira

**Affiliations:** 1 LEPAE, Department of Chemical Engineering, Faculty of Engineering, University of Porto, Rua Roberto Frias, 4200-465, Porto, Portugal; 2 IBB-Institute for Biotechnology and Bioengineering, Centre for Biological Engineering, Universidade do Minho, Campus de Gualtar, 4710-057, Braga, Portugal; 3 Environmental Healthcare Unit, Microbiology Group, School of Biological Sciences, University of Southampton, Bassett Crescent East, Southampton, SO16 7PX, United Kingdom

**Keywords:** Nutrient shock, Osmotic shock, Nutrient stress, Substrate-accelerated death

## Abstract

**Background:**

Plating methods are still the golden standard in microbiology; however, some studies have shown that these techniques can underestimate the microbial concentrations and diversity. A nutrient shock is one of the mechanisms proposed to explain this phenomenon. In this study, a tentative method to assess nutrient shock effects was tested.

**Findings:**

To estimate the extent of nutrient shock effects, two strains isolated from tap water (*Sphingomonas capsulata* and *Methylobacterium sp*.) and two culture collection strains (*E. coli* CECT 434 and *Pseudomonas fluorescens* ATCC 13525) were exposed both to low and high nutrient conditions for different times and then placed in low nutrient medium (R2A) and rich nutrient medium (TSA).

The average improvement (A.I.) of recovery between R2A and TSA for the different times was calculated to more simply assess the difference obtained in culturability between each medium. As expected, A.I. was higher when cells were plated after the exposition to water than when they were recovered from high-nutrient medium showing the existence of a nutrient shock for the diverse bacteria used. *S. capsulata* was the species most affected by this phenomenon.

**Conclusions:**

This work provides a method to consistently determine the extent of nutrient shock effects on different microorganisms and hence quantify the ability of each species to deal with sudden increases in substrate concentration.

## Background

Since the first use of culture media to grow and study bacteria, plating techniques have always been considered as the gold-standard to assess the presence of living microorganisms in a certain environment. Developments occurring during the last 30 years have shown however that these methods are actually underestimating both their numbers and diversity. For instance, application of viability-staining techniques have allowed the identification of “viable but nonculturable bacteria” (VBNC) [reviewed in [1], whereas new species of microorganisms identified by 16 S rRNA gene sequencing still remain uncultured [reviewed in [2].

The VBNC state refers to a condition where cells, once exposed to environmental stress (such as nutrient starvation, elevated or lowered osmotic concentrations, oxygen concentrations and exposure to white light), enter into a dormancy phase and fail to grow on the routine media on which they would normally grow [1,3,4], however, culturability for these cells could be recovered under certain conditions. Despite the lack of cultivability, the VBNC state is of great concerns because cells can display enhanced resistance to antibiotics (mainly due to the low metabolic activity), and retain the virulence properties after resuscitation [4-7].

By the time that the notion of VBNC was first proposed in 1982 [8], efforts to improve the recovery of microorganisms from stressful low-nutrient environments had already began. In 1983, Straškrabová [9] showed that starving aquatic bacteria died on rich media possibly due to a high nutrient shock. One of the keystone papers was published two years later by Reasoner and Geldreich [10]. In their study, a new low-nutrient medium denominated R2A, was presented and found to yield significantly higher bacterial counts than plate count agar for samples from potable water supplies. Similarly, Jensen *et al.*[11] concluded that culturability of specific populations of marine bacteria can be dramatically improved by the use of low-nutrient media. Alternatively, Hahn et al, developed a method - the filtration-acclimatization method - which avoids the nutrient shock by using an acclimatization procedure that provides a slow transition from the low environmental substrate concentrations to the high concentration of standard microbial media. It hence enables the isolation and cultivation (on high nutrient media) of a broad variety of previously uncultured bacteria [12,13]. High-throughput culturing methods that rely on dilution to extinction in very-low-nutrient media or in the step-wise acclimatization to higher substrate concentrations are becoming more common for the isolation of previously uncultured microorganisms, or even novel species, from the ocean, lakes, soils and other low nutrient environments [e. g. [14-18]. All these papers confirmed the earlier suggestions of substrate-accelerated death in bacteria described by Postgate and co-workers [19,20]. More recently, insights into how bacteria adapt their metabolism to these conditions are being provided by molecular biology methods [4,21]. Putting all this together it is acceptable to say that the correct recovery of bacteria is an essential issue on the knowledge of the true roles and function of bacteria in the specific environment.

Based on the success of the previous studies, we have designed a new low-nutrient medium with the specific purpose of a more efficient recovery of the human pathogen *Helicobacter pylori* exposed to water or related environments [22]. In addition, it was concluded that because the direct recovery from water to a high-nutrient medium causes nutrient shock, the bacteria could physiologically adapt to low-nutrient environments and hence be transmitted through water [23].

Even though the existence of a nutrient shock has become engrained in the scientific community, there is no proof of principle for this concept in the literature. Observations such as that R2A is only used for low nutrient environments, or the fact that some freshwater isolates that fail to grow initially in nutrient-rich media may be gradually acclimated to rich media [15], appear to further sustain that notion, but to prove it requires evidence that the ratio of recovery between low and nutrient rich media for a certain microorganism decreases as the microorganism is recovered from low to rich nutrient environments. To the author’s knowledge, this work represents the first attempt to estimate the extent of nutrient shock effects for a range of different bacteria.

## Material and methods

### Recovery and identification of microorganisms from tap water

Two of the microorganisms used for this study were isolated from the drinking water distribution system described by Simões *et al.*[24]. The bacteria were isolated in the planktonic state by plating on R2A (Oxoid, Basingstoke, U.K.) at room temperature during 15 days. Based on 16 S rRNA sequence similarity (performed at the Sequencing and Fragment Analysis Laboratory, Science Faculty of Lisbon) they have been presumptively identified as *Sphingomonas capsulata* (maximum identity of 99%, E value 0.0, with S*phingomonas capsulatum* NR_025838.1 – has been renamed as Novosphingobium capsulatum in [25]) and *Methylobacterium sp.* (maximum identity of 100%, E value 0.0, with *Methylobacterium* sp. AB673245.1) using a Blast search.

### Culture maintenance and media preparation

Culture collection *E. coli* (CECT 434) was maintained in Tryptone Soya Agar (TSA) whereas *Pseudomonas fluorescens* (ATCC 13525) was maintained in Pseudomonas medium prepared as described in Oliveira *et al.*[26]. The water isolates *S. capsulata* and *Methylobacterium sp*. were maintained on R2A. All strains were incubated at 23 ± 2°C and subcultured to new plates every 5 to 7 days.

To assess the existence of a nutrient shock, both a rich nutrient medium (TSA) and a lower nutrient medium (R2A) were used. The final concentration of all constituents in R2A was 18.1 g/L whereas in TSA was 45 g/L. TSA was prepared with 30 g/L of Tryptone Soya Broth (TSB; VWR International, Lisbon, Portugal) and 15 g/L of granulated agar (VWR International). For each set of experiments, both media were prepared and poured into plates seven days before the experiment, and stored at 4°C.

### Exposure of bacteria to low and high-nutrient environments and subsequent recovery

For the experiment in low-nutrient environments, cells from 2 day-old cultures were harvested from R2A plates, suspended in 10 ml of autoclaved tap water, vortexed for 30s and adjusted by optical density to a concentration of 5 × 10^6^ CFU per ml. This inoculum was transferred to a sterile bottle containing 500 ml of autoclaved tap water, to achieve a final concentration of ca. 10^5^ CFU/ml. The bottle was maintained at room temperature (approx. 23 ± 2°C) and continuously stirred (120 rpm) using a magnetic bar. Sampling was performed at different times up to 24 h. Before serial dilution (1:10) in sterile tap water, samples were vortexed for 10s for homogenization. Cells were enumerated in quadruplicate by surface plating 100 μl of the different dilutions onto R2A or TSA. Plates were incubated at 23°C for 7 days and then colony forming units were counted.

For the experiment in high-nutrient environments, the procedure was basically the same, but the water used for the inoculum, bioreactor and dilutions, was replaced by TSB.

### Analysis of data

To more simply assess the difference obtained in culturability between each medium, the average improvement (A.I.) in culturability between R2A and TSA was calculated for all species, both when they were recovered from water and from TSB. The A.I. for each bacterium and condition is defined as follows:

(1) $$A.I.=\frac{\sum_{t=0}^{4}\frac{{\left(\log CFU_{R2A}-\log CFU_{\mathrm{TSA}}\right)}_{t}}{{\left(\log CFU_{\mathrm{TSA}}\right)}_{t}}}{5}\times 100$$

where t represents the different times at which the sampling was performed (0, 2, 4, 6 and 24 h); CFU_R2A_ the colony forming units for each time point on R2A; CFU_TSA_ the colony forming units for each time point on TSA. The formula is divided by 5 as this is the number of sampling times assessed. Based on Equation 1, a nutrient shock index can be calculated:

(2) $$\text{Nutrient shock index }=\text{ A}.\text{I}._{\text{water}}–\text{ A}.\text{I}._{\text{TSB}}$$

where A.I._water_ and A.I._TSB_ represent the average improvement when the cells are cultured from water and TSB, respectively. The larger the nutrient shock index, the more the strains are able to adapt to low nutrient conditions.

Nutrient shock results were statistically analyzed by employing a two-way analysis of variance (ANOVA). Computations were performed using the Statistical Program for the Social Sciences (SPSS Inc., Chicago, USA). Results were considered statistically relevant if P values were ≤0.05.

## Findings

**Figure 1:**
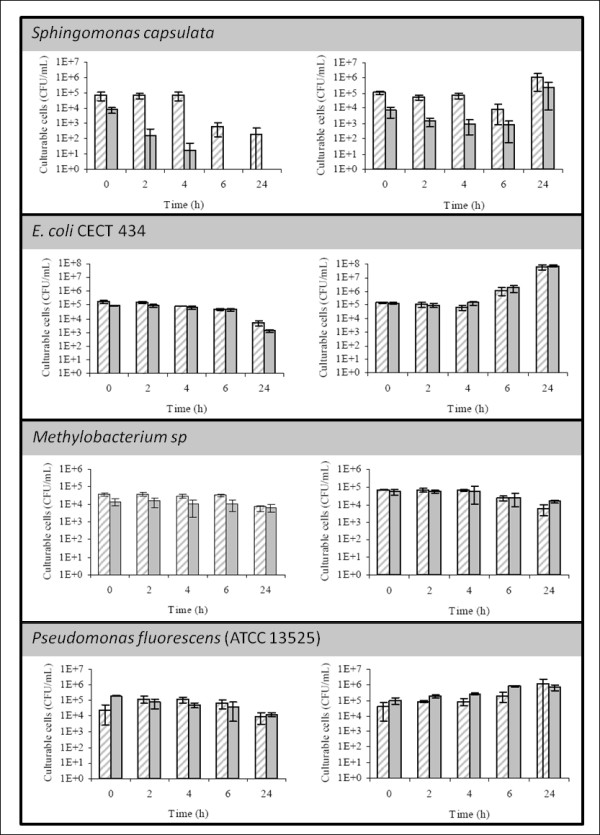
**Study of nutrient shock effect on 4 different microorganisms.** Graphics on the left depict recovery obtained on R2A (dashed bars) and TSA (full bars) as cells are being exposed to water, whereas graphics on the right show recovery when cells are suspended in TSB.

Of the bacteria used for this study, *S. capsulata* appeared to be the one most affected by exposure to water, as after 24 h no cells could be recovered on TSA and only 100 CFU/mL could be recovered in R2A (Figure 1 and Additional file 1 Table A1). In all other bacteria a less noticeable decrease was also observed. When suspended in TSB, *S. capsulata*, *E. coli* and *P. fluorescens* were able to use the nutrients in the media to support growth, whereas *Methylobacterium* sp. lost culturability with time.

For all cases but for *P. fluorescens*, R2A supported better growth for the bacteria recovered from water, a result that was found to be statistically significant for all three cases (Table 1) (P < 0,01). TSA performance improved when the bacteria were recovered from TSB, which implied that differences between recovery in TSA and R2A were no longer statistically significant (P > 0.05). On the other hand, TSA supported a statistically significant higher growth for *P. fluorescens* when the microorganism was recovered from TSB (P < 0.01). In this case, this difference was also attenuated when *P. fluorescens* was recovered from water, and differences between media were no longer statistically significant (P > 0.05).

**Table 1:** Preferred medium for recovering water and TSB exposed strains

	**Recovering from water**	**Recovering from TSB**
*S. capsulata*	R2A^a, c^	R2A
*E. coli* CECT 434	R2A^a^	TSA
*Methylobacterium sp.*	R2A^a^	TSA
*P. fluorescens* ATCC 13525	TSA	TSA^a,b^

^a^ Statistical significant when compared to TSA or R2A values (P < 0.001);

^b^ nutrient diversity of TSA seems to positively affect *P. fluorescens* growth, since TSA performed always better;

^c^ nutrient diversity of R2A seems to positively affect *S. capsulate*, since R2A performed always better.

Reflecting the observations described above, the A.I. was always greater when the bacteria were recovered from water, demonstrating the idea of a nutrient shock, as it can be confirmed by the positive values obtained for all strains in the nutrient shock index (Table 2). Hence, the below-expected performance of TSA for *S. capsulata* and of R2A for *P. fluorescens* is due to intrinsic characteristics of the media (such as nutrient diversity) and not to nutrient concentration differences between the two media.

**Table 2:** Nutrient shock index obtained for the four species used in this study

**Bacterium**	**A.I. in water**	**P value for water**	**A.I. in TSB**	**P value for medium**	**Nutrient shock index**
*S. capsulata*	147^a^	<0.001	38	0.065	109
*E. coli* CECT 434	6.25	<0.001	−1.62	1.000	7.87
*Methylobacterium sp.*	8.71	<0.001	−1.09	1.000	9.78
*P. fluorescens* ATCC 13525	−0.47	1.000	−6.30	<0.001	5.83

^a^For the two time points where readings of CFU for TSA were 0, logCFU was considered to be 1.

Statistical analysis also allowed to observe if the relationship between the recoveries on both media was constant with time. For all cases, the interaction term of time*media was statistically significant (P < 0.05), indicating that this relationship varied. Because the difference between the A.I. for when the cells are recovered from water or from rich-nutrient medium are not very large for three of the microorganisms tested (approx. 7%), the statistical power of the method used was essential to obtain relevant results.

### Nutrient shock occurrence

This study has demonstrated for a set of waterborne microorganisms that a nutrient shock occurs. As an indirect conclusion it shows that newly developed media must not only address the type or types of microorganisms to be recovered but also from where they are being recovered from. Similarly, inferring the adaptability of a certain microorganism to water (or even other environments) based solely on the time it remains culturable might also cause incorrect interpretations as culturability clearly depends on the medium used [27].

As this is a relatively unknown term, it is important to clarify the meaning of nutrient shock and how it correlates with other similar terms such as nutrient stress and osmotic shock. Nutrient stress is a different concept in the sense that it refers to when the quantity of nutrient available decreases yield [28,29]. However, the bacteria might still be in a nutrient rich medium that they are unable to metabolize. Nutrient shock is also different from osmotic stress, as the latter refers only to the physical pressure exerted by the transfer of water through the cell membrane to balance the osmotic pressure [30-32]. Even though part of the nutrient shock effect might be explained by the damage caused by osmotic stress effects on the cells membrane, its broader concept also includes other phenomena such as the inability of the bacteria to suddenly process the large amounts of nutrient of a nutrient-rich solid medium. By the results obtained, it is not possible to understand the contribution that osmotic stress has given to the nutrient shock observed here. To understand the exact extent of this contribution, it will be necessary to develop a new experiment where the bacteria are also exposed to a salt or sugar solution.

The main reason why this study was performed with time was our lack of knowledge about whether this factor would influence the final results. Metabolomics experiments have already shown that the response time in physiological mechanisms of bacteria for an environmental change can be in the order of seconds [33]. However, physiological changes will extend for much longer [34]. In our study, the interaction term of time*media obtained from the statistical analysis was statistically significant for all cases, reflecting the continuous adaptation of the bacteria to the environment. The approach of calculating the A.I. provides therefore a more robust system, that is more representative of the nutrient shock effect for different physiological conditions and not easily affected by the transformations occurring in the population of cells in single time points.

### A.I. indications

This study confirms the long suspected notion that nutrient shock hinders the recovery of low-nutrient adapted microorganisms to rich-nutrient medium [10,11,14,35], and provides a method to quantify those effects in bacteria. It is important to state that the developed measure (A.I.) is dependent on the time intervals at which the samples are taken and that for comparable results to be obtained, the same intervals have to be selected. If comparable results are obtained, than the nutrient shock index can serve as a measure of the ability of different bacteria to withstand nutrient shock effects. In theory it is possible to obtain the nutrient shock index using any other combination of nutrient rich and nutrient poor media other than TSA and R2A, however, once again comparable results might not be obtained.

Future work will involve correlation of A.I. with genome analysis and protein expression in order to shed new light on the mechanisms that are affected by the nutrient shock effect. Another important study would involve the use specific substances used as resuscitation components [3,4] prior to bacteria plating, in order to stimulate bacteria that may be at a VBNC state. This would allow to understand in what extend VBNC cells are correlated with the nutrient shock phenomenon.

## Abbreviations

A.I: Average improvement; VBNC: Viable but nonculturable bacteria; TSA: Tryptone Soya Agar; TSB: Tryptone Soya Broth; SPSS: Statistical Program for the Social Sciences; CFU: Colony forming units.

## Competing interests

The authors declare that they have no competing interests.

## Authors’ contributions

Conceived and designed the experiments: NFA, SMB, CA, LC, CWK, MJV. Performed the experiments: NFA, SMB, LCS. Analyzed the data: CA, NFA, SRBS, LC. Contributed reagents/materials/analysis tools: CWK MJV. Wrote the paper: NFA, SMB, CA. Revised a draft of the manuscript: CWK MJV. All authors read and approved the final manuscript.

## Supplementary Material

Additional files 1 **Table A1 Values of colony forming units (CFU) and respective standard deviation obtained for all species at different times when exposed to water and then plated on R2A and TSA.** Table A2 Values of colony forming units (CFU) and respective standard deviation obtained for all species at different times when exposed to TSB and then plated on R2A and TSA. Click here for file
