# Supplementary material for: Proposal for a method to estimate nutrient shock effects in bacteria
Source: BMC Res Notes. 2012 Aug 8;5:422. doi: 10.1186/1756-0500-5-422 (PMC3490807; doi:10.1186/1756-0500-5-422)
Supplement: Additional files 1 — Table A1 Values of colony forming units (CFU) and respective standard deviation obtained for all species at different times when exposed to water and then plated on R2A and TSA. Table A2 Values of colony forming units (CFU) and respective standard deviation obtained for all species at different times when exposed to TSB and then plated on R2A and TSA. [file 1756-0500-5-422-S1.doc]

Table A1 – Values of colony forming units (CFU) and respective standard deviation obtained for all species at different times when exposed to water and then plated on R2A and TSA.

| Culture medium | Bacteria |  | T=0 | T=2 | T=4 | T=6 | T=24 |
| --- | --- | --- | --- | --- | --- | --- | --- |
| R2A | *Sphingomonas capsulata* | CFU | 7,00E+04 | 6,88E+04 | 7,00E+04 | 6,08E+02 | 1,78E+02 |
|  |  | StDev | 4,00E+04 | 2,63E+04 | 4,00E+04 | 4,68E+02 | 3,42E+02 |
|  | *E. coli* CECT 434 | CFU | 1,63E+05 | 1,43E+05 | 7,80E+04 | 4,60E+04 | 4,67E+03 |
|  |  | StDev | 3,06E+04 | 2,10E+04 | 2,00E+03 | 5,29E+03 | 1,66E+03 |
|  | Methylobacterium sp. | CFU | 3,60E+04 | 3,70E+04 | 3,10E+04 | 3,28E+04 | 7,04E+03 |
|  |  | StDev | 7,09E+03 | 1,10E+04 | 5,20E+03 | 4,27E+03 | 1,68E+03 |
|  | *Pseudomonas fluorescens* | CFU | 2,62E+04 | 1,26E+05 | 1,14E+05 | 6,70E+04 | 9,28E+03 |
|  |  | StDev | 2,37E+04 | 5,89E+04 | 5,07E+04 | 3,85E+04 | 6,47E+03 |
| TSA | *Sphingomonas capsulata* | CFU | 7,75E+03 | 1,70E+02 | 1,75E+01 | 0,00E+00 | 0,00E+00 |
|  |  | StDev | 3,44E+03 | 2,94E+02 | 3,50E+01 | 0,00E+00 | 0,00E+00 |
|  | *E. coli* CECT 434 | CFU | 8,37E+04 | 8,33E+04 | 6,20E+04 | 4,70E+04 | 1,23E+03 |
|  |  | StDev | 6,43E+03 | 2,00E+04 | 1,39E+04 | 1,05E+04 | 3,51E+02 |
|  | Methylobacterium sp. | CFU | 1,48E+04 | 1,50E+04 | 1,05E+04 | 1,08E+04 | 6,32E+03 |
|  |  | StDev | 6,06E+03 | 8,22E+03 | 8,53E+03 | 6,79E+03 | 2,83E+03 |
|  | *Pseudomanas fluorescens* | CFU | 1,92E+05 | 7,30E+04 | 4,98E+04 | 3,93E+04 | 1,30E+04 |
|  |  | StDev | 1,07E+04 | 4,60E+04 | 1,55E+04 | 3,45E+04 | 4,22E+03 |

Table A2 – Values of colony forming units (CFU) and respective standard deviation obtained for all species at different times when exposed to TSB and then plated on R2A and TSA.

| Culture medium | Bacteria |  | T=0 | T=2 | T=4 | T=6 | T=24 |
| --- | --- | --- | --- | --- | --- | --- | --- |
| R2A | Sphingomonas capsulata | CFU | 1,07E+05 | 5,10E+04 | 7,27E+04 | 9,33E+03 | 1,02E+06 |
|  |  | StDev | 2,52E+04 | 1,85E+04 | 3,01E+04 | 8,50E+03 | 8,92E+05 |
|  | E. coli CECT 434 | CFU | 1,43E+05 | 1,01E+05 | 6,80E+04 | 1,14E+06 | 6,10E+07 |
|  |  | StDev | 1,06E+04 | 4,21E+04 | 2,69E+04 | 6,46E+05 | 2,72E+07 |
|  | Methylobacterium sp. | CFU | 7,08E+04 | 6,83E+04 | 6,90E+04 | 2,40E+04 | 5,73E+03 |
|  |  | StDev | 7,76E+03 | 1,40E+04 | 8,29E+03 | 7,12E+03 | 3,41E+03 |
|  | Pseudomonas fluorescens | CFU | 3,75E+04 | 8,48E+04 | 8,73E+04 | 2,04E+05 | 1,08E+06 |
|  |  | StDev | 3,29E+04 | 1,70E+04 | 4,07E+04 | 1,35E+05 | 1,35E+06 |
| TSA | Sphingomonas capsulata | CFU | 7,20E+03 | 1,45E+03 | 9,33E+02 | 8,33E+02 | 2,43E+05 |
|  |  | StDev | 4,98E+03 | 8,21E+02 | 7,51E+02 | 7,77E+02 | 2,35E+05 |
|  | E. coli CECT 434 | CFU | 1,33E+05 | 9,00E+04 | 1,35E+05 | 1,67E+06 | 7,57E+07 |
|  |  | StDev | 2,32E+04 | 2,60E+04 | 4,50E+04 | 8,81E+05 | 1,63E+07 |
|  | Methylobacterium sp. | CFU | 5,75E+04 | 5,48E+04 | 6,00E+04 | 2,50E+04 | 1,53E+04 |
|  |  | StDev | 2,18E+04 | 8,66E+03 | 4,90E+04 | 1,73E+04 | 2,44E+03 |
|  | Pseudomanas fluorescens | CFU | 1,03E+05 | 1,90E+05 | 2,70E+05 | 8,18E+05 | 7,08E+05 |
|  |  | StDev | 4,92E+04 | 3,56E+04 | 5,23E+04 | 6,29E+04 | 2,92E+05 |
